# Heritability of Coronary Artery Disease: Insights From a Classical Twin Study

**DOI:** 10.1161/CIRCIMAGING.121.013348

**Published:** 2022-02-24

**Authors:** Zsofia D. Drobni, Marton Kolossvary, Julia Karady, Adam L. Jermendy, Adam D. Tarnoki, David L. Tarnoki, Judit Simon, Balint Szilveszter, Levente Littvay, Szilard Voros, Gyorgy Jermendy, Bela Merkely, Pal Maurovich-Horvat

**Affiliations:** MTA-SE Cardiovascular Imaging Research Group, (Z.D.D., M.K., J.K., A.L.J., J.S., B.S., P.M.-H.), Semmelweis University, Budapest, Hungary.; Department of Radiology, Medical Imaging Centre (A.D.T., D.L.T., P.M.-H.), Semmelweis University, Budapest, Hungary.; Department of Political Science, Central European University, Budapest, Hungary (L.L.).; Global Genomics Group, Richmond, VA (S.V.).; Bajcsy-Zsilinszky Hospital, Budapest, Hungary (G.J.).; Heart and Vascular Center (B.M.), Massachusetts General Hospital, Harvard Medical School, Boston, MA, USA; Cardiovascular Imaging Research Center, Department of Radiology, Massachusetts General Hospital, Harvard Medical School, Boston, MA, USA (M.K., J.K.).

**Keywords:** atherosclerosis, computed tomography angiography, coronary angiography, coronary artery disease, prevalence

## Abstract

Genetics have a strong influence on calcified atherosclerotic plaques; however, data regarding the heritability of noncalcified plaque volume are scarce. We aimed to evaluate genetic versus environmental influences on calcium (coronary artery calcification) score, noncalcified and calcified plaque volumes by coronary computed tomography angiography in adult twin pairs without known coronary artery disease.

**Methods:**

In the prospective BUDAPEST-GLOBAL (Burden of Atherosclerotic Plaques Study in Twins—Genetic Loci and the Burden of Atherosclerotic Lesions) classical twin study, we analyzed twin pairs without known coronary artery disease. All twins underwent coronary computed tomography angiography to assess coronary atherosclerotic plaque volumes. Structural equation models were used to quantify the contribution of additive genetic, common environmental, and unique environmental components to plaque volumes adjusted for age, gender, or atherosclerotic cardiovascular disease risk estimate and statin use.

**Results:**

We included 196 twins (mean age±SD, 56±9 years, 63.3% females), 120 monozygotic and 76 same-gender dizygotic pairs. Using structural equation models, noncalcified plaque volume was predominantly determined by environmental factors (common environment, 63% [95% CI, 56%–67%], unique environment, 37% [95% CI, 33%–44%]), while coronary artery calcification score and calcified plaque volumes had a relatively strong genetic heritability (additive genetic, 58% [95% CI, 50%–66%]; unique environmental, 42% [95% CI, 34%–50%] and additive genetic, 78% [95% CI, 73%–80%]; unique environmental, 22% [95% CI, 20%–27%]), respectively.

**Conclusions:**

Noncalcified plaque volume is mainly influenced by shared environmental factors, whereas coronary artery calcification score and calcified plaque volume are more determined by genetics. These findings emphasize the importance of early lifestyle interventions in preventing coronary plaque formation.

**Registration:**

URL: https://www.clinicaltrials.gov; Unique identifier: NCT01738828.

**Clinical Perspective**

In a prospective twin study of 196 subjects, coronary computed tomography angiography was performed. Using structural equation models, the heritability of coronary artery calcification score, calcified and noncalcified plaque volumes were estimated. Noncalcified plaque volume was predominantly determined by environmental factors (common environment: 63%, unique environment: 37%), while coronary artery calcification score and calcified plaque volumes had a relatively strong genetic heritability (additive genetic: 58%, unique environmental: 42% and additive genetic: 78%, unique environmental: 22%, respectively). Based on our findings, the genetic background of noncalcified plaque is smaller than that of coronary artery calcification score and calcified plaque. Our results are hypothesis generating but could also imply that socioeconomic status and lifestyle parameters have bigger impact on noncalcified plaque volumes as compared with coronary artery calcification score and calcified plaque volumes. Based on these, there is an urgent need to start primary prevention of coronary artery disease as early in life as possible.

Coronary artery disease (CAD) is a multifactorial disease, influenced by the interplay of environmental and genetic factors.^1^ Heritability of CAD has been estimated to be 40% to 60%, suggesting that genetics play an important role in its development.^2^ Coronary artery calcification (CAC) as assessed by noncontrast computed tomography (CT) has a substantial genetic component, ranging from 30% to 45% in the literature.^3–7^ However, the heritability of noncalcified plaque (NCP) volume has only been addressed in a small number of family studies.^8–10^ Healthy individuals with a family history of early onset CAD have a higher prevalence of NCP.^8^ NCPs are also more prevalent in younger patients with a family history of CAD, compared with patients with no family history^9^ or even to symptomatic patients.^10^ It has been suggested that NCPs represent an earlier stage in atherosclerotic plaque development.^11^ On the contrary, calcification of atherosclerotic plaque may occur in later stages, and calcified plaques (CPs) are less prone to cause events as compared with NCPs.^11,12^

Investigating familial aggregation of complex traits is limited. Shared environment confounds efforts to characterize genetic heritability of complex traits.^13^ However, twins with their precisely matching age represent a unique cohort among family studies,^13^ and they also share a wide range of environmental and socioeconomic variables that may influence the expression of complex traits, such as CAD.^14^ Twin studies by modeling the shared environment, provide a powerful tool to investigate the genetic and environmental factors contributing to a multifactorial disease.^14,15^

Therefore, our aim was to assess the relative contribution of genetic and environmental factors on NCP, CAC score, and CP volumes using coronary CT angiography (CTA) in adult twin pairs without known CAD.

## Methods

The data will be made available from the corresponding author on reasonable request after institutional approval.

### Study Population

This prospective, single-center, classical twin study was conducted under the name of BUDAPEST-GLOBAL (Burden of Atherosclerotic Plaques Study in Twins—Genetic Loci and the Burden of Atherosclerotic Lesions) study.^16^ Participants had been co-enrolled within an international, multicenter clinical study; Genetic Loci and the Burden of Atherosclerotic Lesions (http://www.ClinicalTrials.gov: NCT01738828).^16,17^

Detailed study description and enrollment criteria were published previously.^17^ Monozygotic and same-gender dizygotic twins were recruited from the Hungarian Twin Registry,^18^ all participants provided informed consent. We assessed zygosity using a multiple self-reported questionnaire.^19,20^ The study was approved by the National Scientific and Ethics Committee (institutional review board number: ETT TUKEB 58401/2012/EKU [828/PI/12]) and was performed according to the principles stated in the Declaration of Helsinki.^21^ We excluded 3 twin pairs due to insufficient image quality, resulting in 196 twin subjects as our final cohort.

### Anthropometric Data, Medical History, and Laboratory Analysis

Information regarding past medical history and current medical therapy were recorded by self-reported questionnaires. Fasting peripheral blood draw and brachial blood pressure measurements as well as anthropometric data were recorded before the coronary CTA examination. Fasting blood samples were analyzed by standard methods in a certified laboratory.

### Coronary CTA Image Acquisition and Assessment

Coronary CTA was performed with a 256-slice multidetector CT (Brilliance iCT; Philips HealthTech, Best, the Netherlands).^17^ Prospective ECG triggered noncontrast and contrast-enhanced scans were acquired. Beta-blockers were administered as needed to reach a target heart rate below 60/min. Sublingual nitroglycerin (0.8 mg) was administered on the table. Four-phasic contrast injection protocol was used (Iomeprol 400 g/cm^3^; Iomeron, Bracco Imaging S.p.A., Milan, Italy).^22^ All image analyses were performed offline on a dedicated workstation (Intellispace portal, Philips Healthcare). The coronary CTA data sets were analyzed quantitatively to determine coronary plaque volumes. CAC score was quantified on the noncontrast-enhanced images using the Agatston method.

### Semiautomated Plaque Quantification

**Figure 1.**
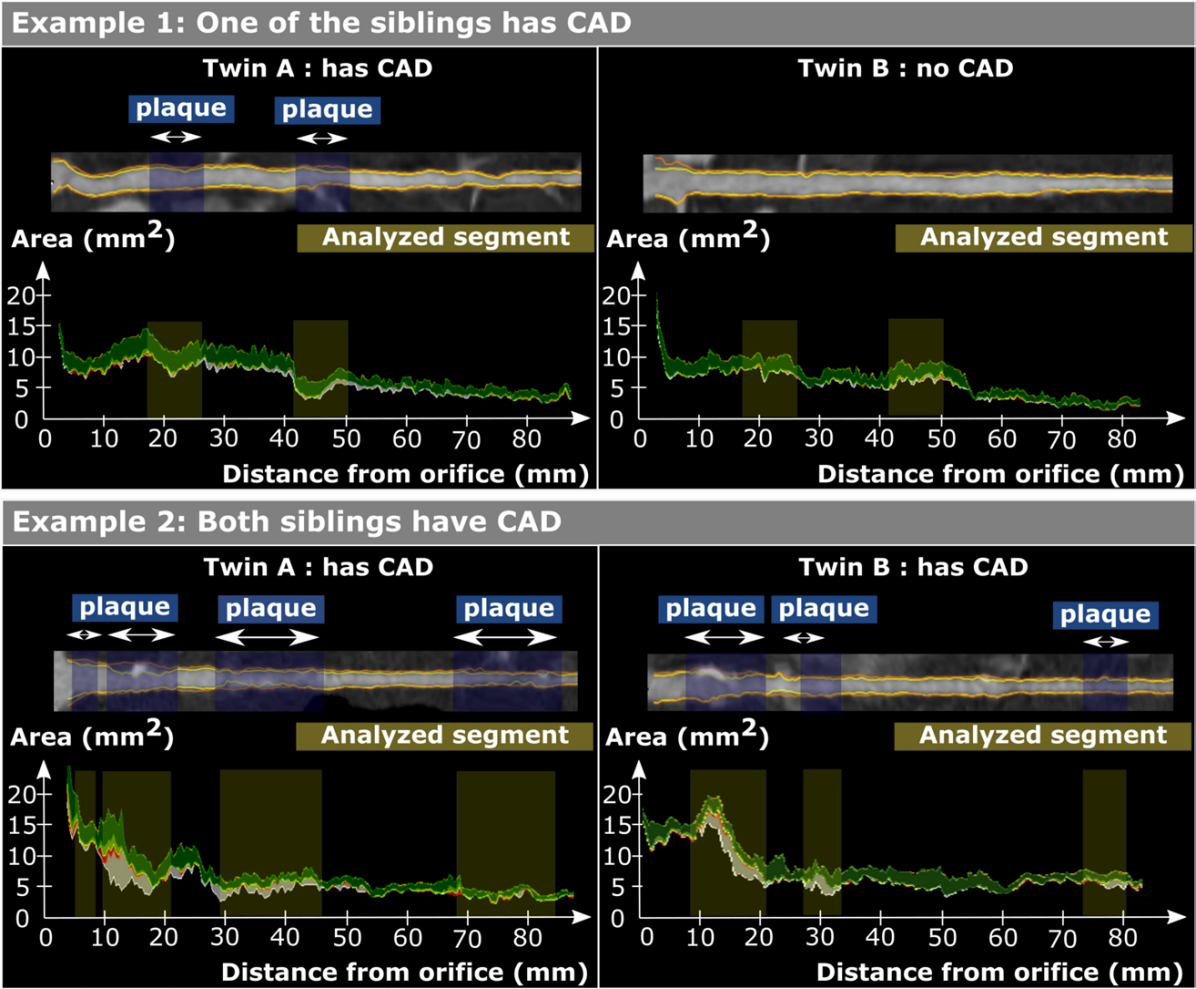
**Demonstration of the plaque analysis method.** Stretched multiplanar reconstruction of the left anterior descending (LAD) artery and quantification graph of the LAD. Yellow shaded regions correspond to analyzed segments and blue shaded regions correspond to plaques. Components of coronary plaque are shown as follows: lipid-rich (low-computed tomographic attenuation) plaque components are shown in red; fibro-fatty tissue is shown in light green; fibrous tissue is shown in dark green; calcium is shown in white. Example 1: One of the siblings had plaques; plaques were analyzed, and for the other sibling, segments in the same location and same length were analyzed. Example 2: Both siblings had plaques; all the segments were analyzed which included any plaque in either of the siblings. In case neither of the siblings had coronary artery disease (CAD), a plaque volume of 0-0 was used.

Quantitative CT analysis was performed by an experienced reader (Dr Drobni) using a dedicated software tool for plaque assessment (QAngio CT; Medis BV, Leiden, the Netherlands; Figure 1). Coronary atherosclerotic plaques were defined as follows: any visible structure that could be assigned to the coronary artery wall in at least 2 independent planes and had a density below the contrast-enhanced coronary lumen but above the surrounding connective tissue. Furthermore, NCP components were defined as a CT density between (−100) HU and 350 HU and CP components as a density between 351 and 2000 HU.^23^ Detailed plaque analysis method is reported in the Supplemental Material.

### Statistical Analysis

Continuous variables are presented as means and SD, while categorical parameters are shown as numbers and percentages. Continuous variables were compared using Student *t* test, whereas binary variables were compared using Fisher exact-test and categorical parameters using the χ^2^ test between monozygotic and dizygotic individuals. Reproducibility of plaque quantification was tested by 2 independent observers (Dr Drobni: 5 years of experience, Dr Simon: 2 years of experience) based on 10 randomly selected monozygotic twin pair and 10 randomly selected dizygotic twin pair images using the intraclass correlation (ICC) coefficient. Coefficient values are interpreted as 1.00 to 0.81: excellent; 0.80 to 0.61: good; 0.60 to 0.41: moderate; 0.40 to 0.21: fair; 0.20 to 0.00: poor. Descriptive statistics, correlations and reproducibility measurements were calculated using IBM SPSS Statistics version 25 (IBM, Armonk, NY).

Genetic structural equation models were built to quantify the proportion of genetic and environmental factors contributing to CAC score, CP, and NCP. These models are based on variation between the twins, which can be broken down to additive genetic factors (A), common environmental factors (C) and unique environmental factors (E), therefore the acronym ACE model.^24^

Additive genetic factors describe the effect of multiple genetic alleles that exert influence additively. Common environmental factors are shared by the twin pairs during their lifetime, such as the same early childhood, education in the same school, living in the same town, sharing similar socioeconomic status even in adulthood, etc.^24^ Unique environmental factors are conditions to which only one of the siblings was exposed.^25^

Sub models of the full models (full ACE model) were calculated, and the more parsimonious model with the best fit was selected. All calculations were adjusted for age and sex or atherosclerotic cardiovascular disease (ASCVD) risk estimate and statin use.^26^ Analysis was performed on the full cohort for CAC and plaque volume (NCP and CP) analysis and on subsets, where siblings with no disease were excluded. Siblings with no disease are referenced as 0-0 pairs. For the 2 different analysis, 2 0-0 subsets were created. For the CAC analysis one subset with those who had a CAC >0 was created, and for the plaque volume analysis another subset, with those who had coronary plaque (either NCP or CP) on the CTA images.

Our raw data on CAC score, NCP and CP were transformed to a log scale, and an inverse-normal transformation on age-sex categories was also performed for the full cohort and for the subsets. Log likelihood-based 95% CI were calculated for all estimated parameters. All analyses were performed using R version 3.5.2.^27^ Twin modeling was performed using OpenMx version 2.12.2. A *P* value of <0.05 was considered statistically significant.

## Results

### Study Population

Baseline demographics and clinical characteristics of the overall population (n=196) are summarized in Table 1. The mean±SD age of the cohort was 56±9 years (63.3% female), and dizygotic subjects were older than the monozygotic subjects (58±8 versus 55±10 years, *P*=0.005). The most prevalent cardiovascular risk factor in the total cohort was hypertension (83/196, 42.3%), and 16.8% (33/196) of the participants were on primary preventive statin therapy (Table 1). Both total cholesterol (214.8±42.2 mg/dL) and LDL (low-density lipoprotein)-cholesterol levels (134.7±38.4 mg/dL) were slightly elevated with no difference between monozygotic and dizygotic groups (*P*=0.25 and *P*=0.35, respectively). The 10-year ASCVD risk estimate was 7.9±7.7% for the total cohort, with 83 subjects as low-risk (<5.0%), 34 subjects as borderline risk (5.0%–7.4%), 63 subjects as intermediate risk (7.5%–19.9%), and 16 high risk (>20.0%) subjects. Significant difference was observed in the HbA1c levels between the monozygotic and dizygotic group (5.6±1.0% versus 5.3±0.8%, *P*=0.01). Otherwise, there were no significant differences between the groups (Table 1).

**Table 1.**
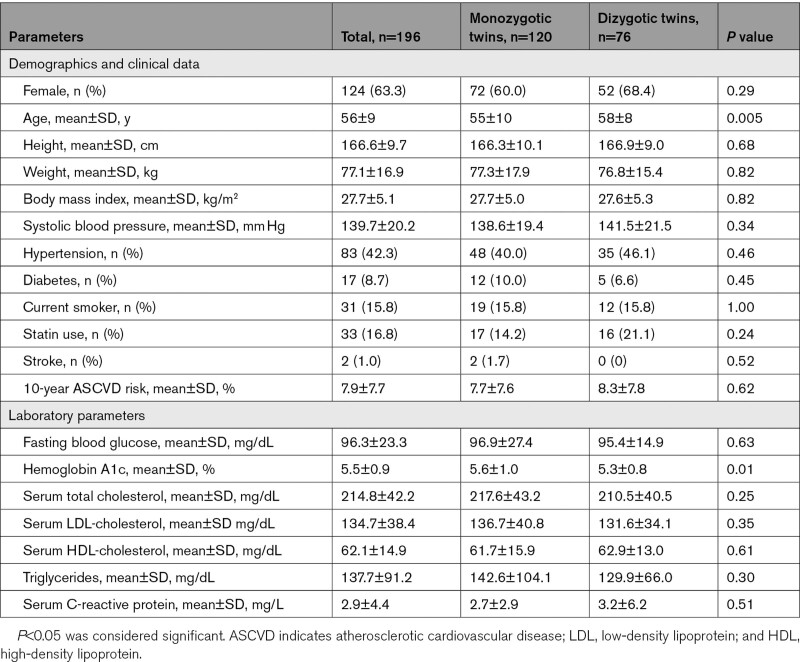
Basic Demographics and Clinical-Laboratory Characteristics of Twin Subjects

Data regarding family history, socioeconomic status, and lifestyle parameters were available in all subjects, and these results are summarized in Table 2. Dizygotic twins had a stronger family history for dyslipidemia (65.8% [50/76] versus 36.7% [44/120], *P*<0.001) and for coronary revascularization (15.8% [12/76] versus 6.7% [8/120], *P*=0.04) as compared with monozygotic twins.

**Table 2.**
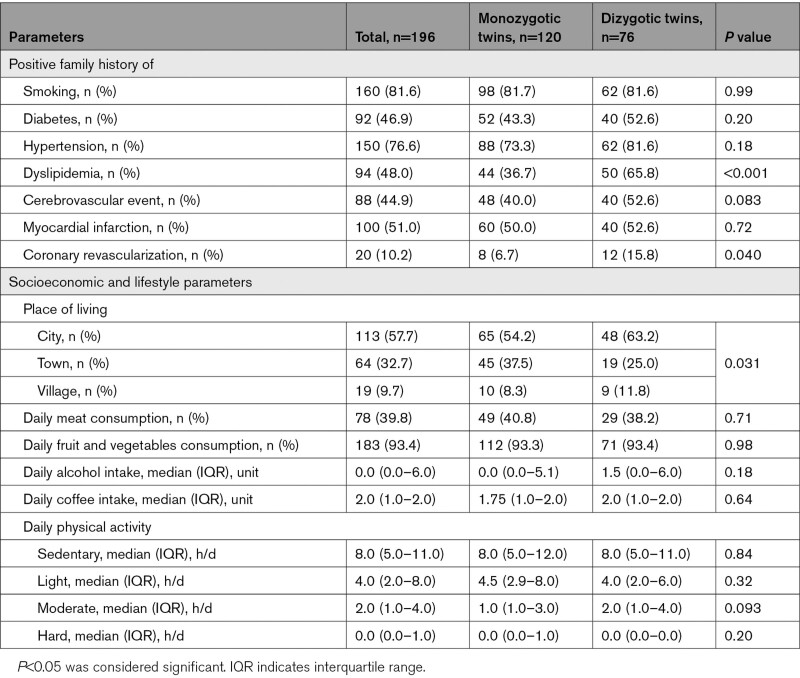
Data on Family History and Lifestyle Parameters

### CAD Characteristics

Intrareader and interreader agreement showed excellent reproducibility for all plaque volume measurements (ICC_intra-reader_ for NCP and CP was 0.99 and 0.98, and ICC_inter-reader_ for NCP and CP was 0.97 and 0.99, respectively).

Out of the 196 twins, 74 (37.8 %) had coronary calcification on noncontrast enhanced images, 34 were dizygotic and 40 were monozygotic. The prevalence of a CAC score higher than zero was not different among the monozygotic and dizygotic twins (44.7% [34/76] versus 33.3% [40/120] *P*=0.13). The prevalence of discordant twin pairs, meaning one sibling had a CAC score higher than zero, whereas the other did not, was significantly higher in the dizygotic group (Table 3). The prevalence of concordant twin pairs with a CAC score of 0 was higher in the monozygotic group (Table 3). Comparing monozygotic and dizygotic twins, we found no differences regarding the median CAC score values in those who had a CAC score higher than 0 (115 [8–336] for the monozygotic group versus 97 [47–208] for the dizygotic group; *P*>0.9).

**Table 3.**
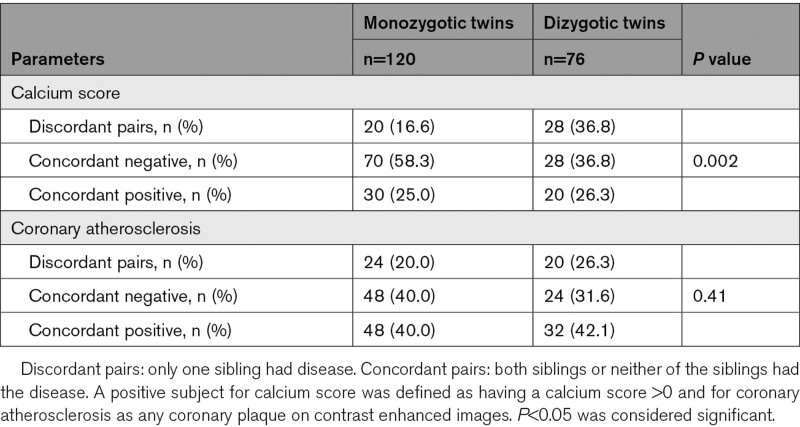
Data on Coronary Artery Disease Characteristics

Out of the 196 twins, 102 (52.0%) had coronary plaques, 42 were dizygotic and 60 were monozygotic. The prevalence of any CAD was not different among the groups. (dizygotic, 55.3% [42/76] versus monozygotic, 50.0% [60/120] group *P*=0.56). In those, who had CAD, the median segment involvement score was 3 (2–5), and the median segment stenosis score was 4 (2–8), with no significant difference among the dizygotic and monozygotic groups (*P*=0.2 for both).

Comparing monozygotic and dizygotic twins with CAD, we found no differences regarding NCP volume (dizygotic, 107 [52–178] mm^3^ versus monozygotic, 79 [36–175] mm^3^; *P*=0.5) and CP volume (dizygotic, 43 [7–65] mm^3^ versus monozygotic, 18 [5–84] mm^3^, *P*=0.4).

### Heritability of CAC Score, CP, and NCP

**Figure 2.**
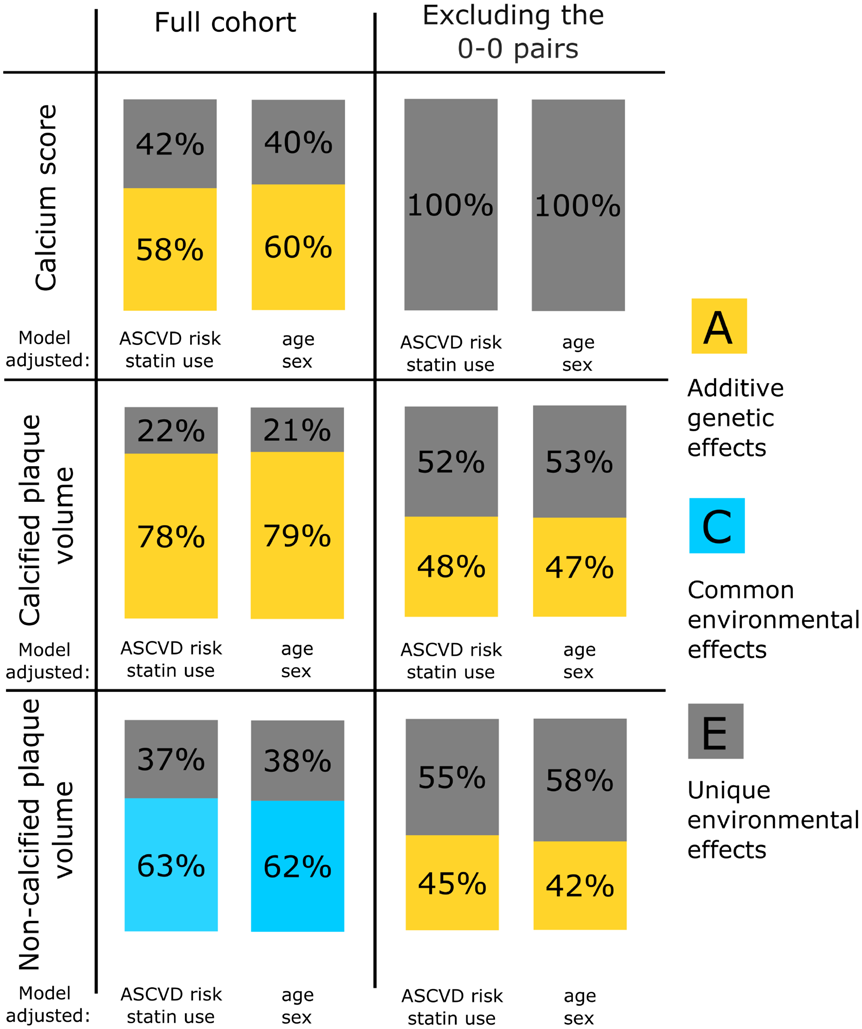
**Twin statistics results.** Bar graphs demonstrate the results of the genetic structural equation model of calcium score, calcified, and noncalcified plaque volumes in both the total cohort and in the 2 subsets of siblings with 0-0 pairs excluded. ASCVD indicates atherosclerotic cardiovascular disease.

Using genetic structural equation models, adjusted for age and gender, ASCVD risk estimate and statin use, the best fitting models were selected both for the full cohort and for the 2 subsets (Figure 2). Twin statistics were performed on the raw data, log transformed and on the inverse normal transformed data. Here, we report the results from the inverse normal transformed data. All the other results can be found in the Supplemental Material.

In the full cohort, CAC score had a strong genetic heritability (adjusted for ASCVD risk and statin use; A: 58% [95% CI, 50%–66%], E: 42% [95% CI, 34%–50%], Table S1 and S2) similarly to CP (adjusted for ASCVD risk and statin use; A: 78% [95% CI, 73%–80%], E: 22% [95% CI, 20%–27%], Tables S3 and S4; Figure 2). On the contrary, NCP quantities were influenced predominantly by common, shared environmental factors (adjusted for ASCVD risk and statin use; C: 63% [95% CI, 56%–67%], E: 37% [95% CI, 33%–44%], Table S5 and S6; Figure 2). Similar results were found when the genetic structural equation models were adjusted for age and gender (Tables S1 through S6).

We repeated the analysis in the subsets of siblings, where both twins without disease were excluded. This resulted in 98 subjects for CAC score, and in 124 subjects for plaque volume (CP and NCP) analysis. Adjusting for ASCVD risk and statin use, the best fitting model was model E, for CAC score showing that 100% of the phenotypic variance in CAC score was attributable to unique environmental factors (E: 100% [95% CI, 100%–100%]). In the subset analysis, the heritability of CP and NCP was similar. In both cases the best fitting model was AE with a modest genetic influence (CP; A: 48% [95% CI, 41%–55%], E: 52% [95% CI, 44%–59%], Table S4 and NCP; 45% [95% CI, 36%–52%], E: 55% [95% CI, 48%–64%], Table S6).

## Discussion

Using coronary CTA in adult twins without known CAD, we found that NCP volume was mostly influenced by environmental, rather than genetic factors, while CAC score and CP volume had a relatively strong genetic heritability. Our results remained similar, irrespective of the adjusted covariates. We repeated our analysis in 2 cohorts where the 0-0 pairs were excluded. The contribution of genetic factors to phenotypic variance of CAC score disappeared and diminished for CP from 78% to 48%. The contribution of common, shared environmental factors to the phenotypic variance of NCP disappeared, and a moderate genetic background of 45% was found when the 0-0 pairs were excluded. The loss of a genetic contribution in the subgroup analysis for CAC score could also suggest that the overwhelming majority of genetic contributions are protective against coronary calcification.

Positive family history of CAD is considered to be an independent risk factor for future cardiovascular events.^28^ In the Framingham Heart Study, a positive family history of CAD was a strong predictor of CAD^29,30^ and was the strongest clinical predictor in younger patients of future myocardial infarction.^9^ Healthy first-degree relatives of patients with early onset CAD have an ≈5-fold increase of total coronary plaque volume compared with symptomatic patients.^10^ It has been demonstrated that CAC has a substantial genetic component.^3–7^ In line with these findings, the investigators of a community-based study from Rochester reported that >40% of the interindividual variation in CAC quantity is attributed to genetic factors.^3^ These findings suggest a strong hereditary component of coronary atherosclerosis. In our study, we found a slightly stronger genetic background both for CAC score (58%) and CP (78%) as previously reported in the above-mentioned studies.

The Swedish twin trial enrolled over 20 000 twins, and the genetic basis of CAD-related mortality was evaluated. An increased risk for CAD was found among close relatives.^1,2^ If a twin sibling had died from early onset CAD, the sibling’s relative hazard of death due to CAD was roughly double in male monozygotic twins as compared with male dizygotic twins, and nearly 6× higher in females.^1^ Among our twin subjects, there were more discordant pairs for CAC score among the dizygotic group and more concordant pairs among the monozygotic groups. These results also imply a strong genetic background of coronary calcification as also shown in more detail with the genetic structural models in our main analysis.

In our classical twin study, we found a relatively strong genetic dependence of CAC score and CP volumes, corroborating former observations.^3,7^ On the contrary, we observed that environment, especially common environmental factors play a more important role than genetic effects in determining NCP volumes in individuals without known CAD. These findings are seemingly in contradiction to former suggestions, which describe the importance of genetics in CAD development.^8,10^ However, the fact that a trait runs in family does not mean that it has a strong genetic background, since families may share not only genes but common environmental factors, for example socioeconomic status, dietary, and exercise habits.^13,24^ In a family study, distinction between genetic and common environmental effects may be difficult,^13,31,32^ but twin studies can overcome this limitation, since twin studies can estimate the magnitude of genetic and common environmental components to a specific trait, by modeling the shared environment.^13,15,33^ This may be a reason why we only found a strong genetic component behind NCP volumes after adjusting for age and gender or ASCVD risk estimate and statin use and only in a subset of twins when we excluded the 0-0 pairs. Importantly, twin studies, compared with family studies, can be more powerful to examine heritability of a complex trait because they can take advantage of a unique characteristics of twins: their shared genetics and matching age.^15^ Furthermore, a twin design naturally accounts for maternal factors and a range of early environmental factors, which might potentially bias the associations.^14^ Twins are exposed to higher degree of shared family environment compared with nontwin siblings, and they also share a range of environmental variables and socioeconomic status even in adulthood,^32^ which do contribute to the expression of complex traits.^33^ Therefore, we believe that our findings are complimentary and additive to previous observations regarding the importance of genetic and environmental factors influencing plaque volumes.

Our results did change when we analyzed CAD heritability in a subgroup, excluding the 0-0 plaque pairs. Since this study enrolled asymptomatic subjects with no history of CAD, excluding the 0-0 pairs did significantly reduce our sample size. Performing twin statistics on a phenotype which can be 0 is extremely challenging, and we approached it from several angles. In cases where one sibling had CAD and the other did not, we co-registered the plaque localization and used that instead of 0 for the nondiseased sibling. Moreover, we performed a rank based inverse normal transformation, to balance out the 0 tail in the data distribution caused by the nondiseased subjects. The inverse normal transformation removed the heavy 0 tail, and we further adjusted it for age, sex, and ASCVD risk estimate and statin use.

It is important to note that our findings do not explain the underlying pathophysiological background of CAD and plaque formation. Nevertheless, it is generally accepted that during coronary plaque formation, NCPs are present at early stage while CPs represent a later stage of plaque development.^11^ Accordingly, our results indicate that early development of coronary plaques is mainly influenced by common environmental factors, such as dietary and exercise habits and socioeconomic status, in contrast to plaque calcification which is more dependent on genetics.

An important clinical implication of our findings is within primary prevention of CAD, especially of NCP. Based on our findings, the genetic background of NCP is smaller than the CAC score and CP, and our data also imply that socioeconomic status and lifestyle parameters have bigger impact on NCP as compared with CAC score and CP. Based on these, there is an urgent need to start primary prevention of CAD as early in life as possible. Starting with lifestyle changes could be the foundation of preventative measures for NCP. The data and analysis presented in this paper should be considered hypothesis-generating and may need further confirmation.

### Limitations

Our sample size is relatively modest and comparable with other twin studies.^34^ The aim was to balance the overall population for 50% females and ≥50% dizygotic twins; however, 63% of the twins are female and 39% are dizygotic twins. This might be due to the fact that females and monozygotic twins are more willing to participate in research than males.^35^ In the present study, statins were used in 16.8% of the patients, which is relatively low; however, it can still influence the presence and phenotype of CAD.^36^ Measurement error may appear as part of the unique environmental component as it is uncorrelated across measurements. Due to the cross-sectional nature of our study, we had no data about the plaque development over time. The study was a single-center investigation with twins from a White population, which may limit the generalizability of our findings. A potential bias could be the age difference between the monozygotic and dizygotic groups, which could inflate the correlation among the dizygotic group, especially for CP volumes. However, calcified and NCP volumes were not different among the monozygotic and dizygotic groups. We believe that by performing the heritability analysis on the inverse-normal transformed data on age-sex categories and adjusting for age and gender and for ASCVD risk estimate, we were able to address this issue.

## Conclusions

We have observed that NCP volume is predominantly determined by common environmental factors while CAC score and CP volume are influenced mainly by genetics. These findings suggest that lifestyle may have an important role in the initiation of CAD (NCP), and genetics may have a strong effect on CP formation. These results underline the importance of optimal risk factor management early in life.

## Article Information

### Acknowledgments

We thank the team at the Heart and Vascular Center for their help in performing the study.

### Sources of Funding

This study was supported by a New Horizons Grant from the EASD to Dr Jermendy. Dr Drobni was supported by the ÚNKP-21-4-I-SE new national excellence program of the ministry for innovation and technology from the source of the national research, development, and innovation fund. Dr Merkely was funded by Project no. NVKP_16-1–2016-0017 (National Heart Program) with the support provided from the National Research, Development and Innovation Fund of Hungary, financed under the NVKP_16 funding scheme. The research was financed by the Thematic Excellence Programme (2020-4.1.1.-TKP2020) of the Ministry for Innovation and Technology in Hungary, within the framework of the Therapeutic Development and Bioimaging thematic programmes of the Semmelweis University.

### Disclosures

Dr Voros is a shareholder in Global Genomics Group, LLC, and receives salary from Global Genomics Group, LLC. The other authors report no conflicts.

### Supplemental Materials

Supplemental Document

Tables S1–S6

## Supplementary Material


