# Supplementary material for: Heritability of Coronary Artery Disease: Insights From a Classical Twin Study
Source: Circ Cardiovasc Imaging. 2022 Feb 24;15(3):e013348. doi: 10.1161/CIRCIMAGING.121.013348 (PMC8925867; doi:10.1161/CIRCIMAGING.121.013348)
Supplement: Supplementary file 1 [file hci-15-e013348-s001.pdf]

SUPPLEMENTAL MATERIAL

This appendix has been provided by the authors to give readers additional information about their work.

Supplement to: **Heritability of coronary artery disease: insights from a classical twin study**

Zsofia D. Drobni, MD, PhD[a], Marton Kolossvary, MD, PhD[a], Julia Karady, MD[a], Adam L. Jermendy, MD, PhD[a], Adam D. Tarnoki, MD, PhD[b], David L. Tarnoki, MD, PhD[b], Judit Simon, MD[a], Balint Szilveszter, MD, PhD[a], Levente Littvay, PhD[c], Szilard Voros, MD, PhD[d], Gyorgy Jermendy, MD, PhD[e], Bela Merkely, MD, PhD[a], Pal Maurovich-Horvat, MD, PhD, MPH [a,b]

[a]MTA-SE Cardiovascular Imaging Research Group, Heart and Vascular Center, Semmelweis University, Budapest, Hungary

[b]Department of Radiology, Medical Imaging Centre, Semmelweis University, Budapest, Hungary

[c]Department of Political Science, Central European University, Budapest, Hungary

[d]Global Genomics Group, Richmond, VA; United States of America

[e]Bajcsy-Zsilinszky Hospital, Budapest, Hungary

Table of Contents

Document S1: Semiautomated plaque quantification and co-registration.............................. 3

Table S1: Detailed model information regarding single trait classical twin model of Calcium score (n = 196). The most parsimonious models are bolded. 4

Table S2: Detailed model information regarding single trait classical twin model of Calcium score excluding the 0-0 pairs (n = 98). The most parsimonious models are bolded. 5

Table S3: Detailed model information regarding single trait classical twin model of calcified plaque volume (n = 196). The most parsimonious models are bolded.................................... 6

Table S4: Detailed model information regarding single trait classical twin model of calcified plaque volume excluding the 0-0 pairs (n = 124). The most parsimonious models are bolded.. 7

Table S5: Detailed model information regarding single trait classical twin model of non-calcified plaque volume (n = 196). The most parsimonious models are bolded....................... 8

Table S6: Detailed model information regarding single trait classical twin model of non-calcified plaque volume excluding the 0-0 pairs (n = 124). The most parsimonious models are bolded... 9

Document S1: Semiautomated plaque quantification and co-registration.

First, automated segmentation of vessel and lumen border was performed for the main epicardial vessels and for the side branches, with ≥2 mm in luminal diameter. Data sets were evaluated for the presence of any coronary atherosclerotic plaques. Coronary atherosclerotic plaques were defined as follows: any visible structure that could be assigned to the coronary artery wall in at least two independent planes and had a computed tomography density below the contrast-enhanced coronary lumen but above the surrounding connective tissue. Tracings were manually corrected when necessary. In the second step, the proximal and distal edges of the plaques were marked manually using multiplanar coronary CTA images. In the third step, twin-pair-based co-registration was performed:[17,23]

1) If one of the twins had plaques but the other did not, then we matched the plaques in location (using the distance from the orifice or from bifurcations) and length from the diseased sibling to the healthy segment. Same length segments in the same location were analyzed for the healthy sibling as for the diseased one to adjust for vessel wall volume (Example 1 on Figure 1).

2) If both siblings had CAD, then all the plaques were analyzed for both siblings (Example 2 on Figure 1).

3) If none of the siblings had coronary plaque, then 0 was used.

Table S1: Detailed model information regarding single trait classical twin model of Calcium score (n = 196). The most parsimonious models are bolded.

Adjusted for		Estimated parameters	A[*]	95% CI	C[†]	95% CI	[‡]E	95% CI	Model *-2LL*	df	AIC	Difference to full model *-2LL*	Difference to full model *p*
**Calcium score - raw data**													
Age	Sex	ACE	49.75	43.49-74.01	0.00	0.00-29.66	50.25	28.23-56.56	508.87	190.00	128.87		
		**AE**	**49.75**	**43.97-70.04**			**50.25**	**29.96-56.03**	**508.87**	**191.00**	**126.87**	**0.00**	**1.00**
		CE			34.59	30.95-40.73	65.41	59.27-69.05	512.23	191.00	130.23	3.36	0.07
		E					100	100-100	524.72	192.00	140.72	15.85	0.00
ASCVD§ Risk	Statin	ACE	40.82	13.66-62.76	3.05	0.00-23.38	56.13	38.62-66.73	509.07	190.00	129.07		
		**AE**	**44.47**	**36.65-60.12**			**55.53**	**39.88-65.35**	**509.08**	**191.00**	**127.08**	**0.01**	**0.91**
		CE			31.22	27.65-36.38	68.78	63.62-72.35	510.42	191.00	128.42	1.35	0.25
		E					100	100-100	520.17	192.00	136.17	11.10	0.00
**Calcium score - log transformed data**													
Age	Sex	ACE	75.59	69.52-81.46			24.41	18.54-30.48	441.81	190.00	61.81		
		**AE**	**75.59**	**69.41-80.80**			**24.41**	**19.02-30.59**	**441.81**	**191.00**	**59.81**	**0.00**	**1.00**
		CE			45.94	39.32-52.52	54.06	47.48-60.68	463.13	191.00	81.13	21.33	0.00
		E					100	100-100	486.36	192.00	102.36	44.56	0.00
ASCVD§ Risk	Statin	ACE	76.87	70.82-83.56			23.13	16.97-29.32	445.34	190.00	65.34		
		**AE**	**76.87**	**70.05-82.01**			**23.13**	**17.99-29.95**	**445.34**	**191.00**	**63.34**	**0.00**	**1.00**
		CE			48.17	40.67-54.30	51.83	45.70-59.33	465.53	191.00	83.53	20.20	0.00
		E					100	100-100	490.35	192.00	106.35	45.01	0.00
**Calcium score - inverse normal transformed data**													
Age	Sex	ACE	59.87	58.11-71.35			40.13	29.22-41.97	531.14	190.00	151.14		
		**AE**	**59.87**	**56.75-69.15**			**40.13**	**30.85-43.25**	**531.14**	**191.00**	**149.14**	**0.00**	**1.00**
		CE			36.48	32.25-45.52	63.52	54.48-67.75	541.02	191.00	159.02	9.88	0.00
		E					100	100-100	555.01	192.00	171.01	23.87	0.00
ASCVD§ Risk	Statin	ACE	57.92	49.42-65.61			42.08	35.21-50.95	527.95	190.00	147.95		
		**AE**	**57.92**	**50.25-65.73**			**42.08**	**34.27-49.75**	**527.95**	**191.00**	**145.95**	**0.00**	**1.00**
		CE			34.63	27.44-41.76	65.37	58.24-72.56	537.07	191.00	155.07	9.12	0.00
		E					100	100-100	548.89	192.00	164.89	20.94	0.00

[*]A: additive genetic factor, [†]C: common environmental factor, [‡]E: unique environmental factor, §ASCVD: Atherosclerotic Cardiovascular Disease

Table S2: Detailed model information regarding single trait classical twin model of Calcium score excluding the 0-0 pairs (n = 98). The most parsimonious models are bolded.

Adjusted for	Estimated parameters	A*	95% CI	C†	95% CI	‡E	95% CI	Model *-2LL*	df	AIC	Difference to full model *-2LL*	Difference to full model *p*
**Calcium score - raw data**												
Age Sex	ACE	27.93	12.45-52.33	5.96	0.00-17.41	66.11	50.0-73.1	262.06	92.00	274.06		
	**AE**	**35.04**	**27.98-51.09**			**64.96**	**48.91-72.02**	**262.08**	**93.00**	**272.08**	**0.02**	**0.88**
	CE			26.05	22.49-33.55	73.95	66.45-77.51	262.35	93.00	272.35	0.29	0.59
	E					100	100-100	265.79	94.00	273.79	3.73	0.15
ASCVD§ Risk Statin	ACE	9.22	0.00-44.79	19.10	12.09-29.35	71.68	59.78-79.75	263.47	92.00	275.47		
	AE	32.90	25.6-47.58			67.10	52.42-74.40	263.68	93.00	273.68	0.21	0.64
	**CE**			**25.49**	**21.16-32.20**	**74.51**	**67.80-78.84**	**263.50**	**93.00**	**273.50**	**0.03**	**0.87**
	E					100	100-100	266.69	94.00	274.69	3.22	0.20
**Calcium score - log transformed data**												
Age Sex	ACE	37.88	28.97-56.29			62.12	43.71-71.03	257.05	92.00	269.05		
	**AE**	**37.88**	**26.29-53.35**			**62.12**	**46.65-73.71**	**257.05**	**93.00**	**267.05**	**0.00**	**1.00**
	CE			12.97	6.09-23.88	87.03	76.12-93.91	259.91	93.00	269.91	2.86	0.09
	E					100	100-100	260.74	94.00	268.74	3.69	0.16
ASCVD§ Risk Statin	ACE	45.44	35.37-60.70			54.56	39.23-64.66	256.89	92.00	268.89		
	**AE**	**45.44**	**35.94-60.58**			**54.56**	**39.42-64.06**	**256.89**	**93.00**	**266.89**	**0.00**	**1.00**
	CE			18.44	10.59-28.20	81.56	71.80-89.41	260.49	93.00	270.49	3.60	0.06
	E					100	100-100	262.16	94.00	270.16	5.27	0.07
**Calcium score - inverse normal transformed data**												
Age Sex	ACE	14.62	12.01-31.32			85.38	69.13-88.25	276.50	92.00	288.50		
	AE	14.62	12.20-30.65			85.38	69.35-87.80	276.50	93.00	286.50	0.00	1.00
	CE			5.55	3.22-17.27	94.45	82.73-96.78	276.95	93.00	286.95	0.45	0.50
	**E**					**100**	**100-100**	**277.10**	**94.00**	**285.10**	**0.60**	**0.74**
ASCVD§ Risk Statin	ACE	12.83	6.23-27.25	0.00	0.00-23.73	87.17	74.27-94.45	275.13	92.00	287.13		
	AE	12.83	3.55-24.66			87.17	75.34-96.45	275.13	93.00	285.13	0.00	1.00
	CE			4.14	0.00-14.77	95.86	85.23-100	275.50	93.00	285.50	0.37	0.54
	**E**					**100**	**100-100**	**275.58**	**94.00**	**283.58**	**0.45**	**0.80**

*A: additive genetic factor, †C: common environmental factor, ‡E: unique environmental factor, §ASCVD: Atherosclerotic Cardiovascular Disease

Table S3: Detailed model information regarding single trait classical twin model of calcified plaque volume (n = 196). The most parsimonious models are bolded.

Adjusted for	Estimated parameters	A*	95% CI	C†	95% CI	‡E	95% CI	Model *-2LL*	df	AIC	Difference to full model *-2LL*	Difference to full model *p*
**Calcified plaque volume - raw data**												
Age Sex	ACE	62.82	56.78-77.51			37.18	22.49-43.22	491.06	190.00	111.06		
	**AE**	**62.82**	**58.86-78.11**			**37.18**	**21.89-41.14**	**491.06**	**191.00**	**109.06**	**0.00**	**1.00**
	CE			43.35	40.03-49.02	56.65	50.98-59.97	498.02	191.00	116.02	6.96	0.01
	E					100	100-100	518.42	192.00	134.42	27.35	0.00
ASCVD§ Risk Statin	ACE	56.05	43.63-76.10	0.16	0.00-19.54	43.78	26.61-52.19	492.99	190.00	112.99		
	**AE**	**56.24**	**47.47-70.49**			**43.76**	**29.51-52.53**	**492.99**	**191.00**	**110.99**	**0.00**	**0.99**
	CE			39.22	35.75-43.64	60.78	56.36-64.25	496.21	191.00	114.21	3.22	0.07
	E					100	100-100	511.93	192.00	127.93	18.94	0.00
**Calcified plaque volume - log transformed data**												
Age Sex	ACE	73.75	58.12-86.92	17.06	2.67-33.27	9.18	7.45-10.29	351.62	190.00	-28.38		
	**AE**	**90.72**	**89.64-92.41**			**9.28**	**7.59-10.36**	**352.26**	**191.00**	**-29.74**	**0.64**	**0.42**
	CE			74.73	71.07-78.80	25.27	21.2-28.93	383.46	191.00	1.46	31.84	0.00
	E					100	100-100	463.58	192.00	79.58	111.97	0.00
ASCVD§ Risk Statin	**ACE**	**56.56**	**43.60-72.48**	**34.51**	**19.06-47.01**	**8.94**	**7.74-10.94**	**377.24**	**190.00**	**-2.76**		
	AE	90.68	88.9-92.09			9.32	7.91-11.1	379.92	191.00	-2.08	2.68	0.10
	CE			77.34	73.04-80.71	22.66	19.29-26.96	399.86	191.00	17.86	22.63	0.00
	E					100	100-100	479.24	192.00	95.24	102.00	0.00
**Calcified plaque volume - inverse normal transformed data**												
Age Sex	ACE	62.93	44.56-75.56	16.17	0.00-35.40	20.90	15.63-22.31	490.24	190.00	110.24		
	**AE**	**79.29**	**77.8-84.15**			**20.71**	**15.85-22.2**	**490.72**	**191.00**	**108.72**	**0.49**	**0.49**
	CE			65.12	61.60-70.79	34.88	29.21-38.4	501.09	191.00	119.09	10.86	0.00
	E					100	100-100	555.16	192.00	171.16	64.92	0.00
ASCVD§ Risk Statin	ACE	56.16	39.21-72.26			21.07	4.04-38.04	485.91	190.00	105.91		
	**AE**	**77.60**	**73.22-80.29**			**22.40**	**19.71-26.78**	**486.73**	**191.00**	**104.73**	**0.82**	**0.37**
	CE			64.26	58.56-68.05	35.74	31.95-41.44	493.91	191.00	111.91	8.00	0.00
	E					100	100-100	543.95	192.00	159.95	58.04	0.00

*A: additive genetic factor, †C: common environmental factor, ‡E: unique environmental factor, §ASCVD: Atherosclerotic Cardiovascular Disease

Table S4: Detailed model information regarding single trait classical twin model of calcified plaque volume excluding the 0-0 pairs (n = 124). The most parsimonious models are bolded.

Adjusted for	Estimated parameters	A[*]	95% CI	C[†]	95% CI	[‡]E	95% CI	Model *-2LL*	df	AIC	Difference to full model *-2LL*	Difference to full model *p*
**Calcified plaque volume - raw data**												
Age Sex	ACE	55.49	50.25-76.38			44.51	25.04-49.30	319.92	118.00	83.92		
	**AE**	**55.49**	**50.45-72.64**			**44.51**	**27.36-49.55**	**319.92**	**119.00**	**81.92**	**0.00**	**1.00**
	CE			37.29	33.67-43.37	62.71	56.63-66.33	322.83	119.00	84.83	2.92	0.09
	E					100	100-100	332.11	120.00	92.11	12.20	0.00
ASCVD§ Risk Statin	ACE	52.69	39.68-71.91	0.00	0.00-28.00	47.30	30.42-55.47	323.30	118.00	87.30		
	**AE**	**52.69**	**43.16-66.67**			**47.31**	**33.33-56.84**	**323.30**	**119.00**	**85.30**	**0.00**	**1.00**
	CE			36.74	33.04-40.13	63.26	59.87-66.96	325.08	119.00	87.08	1.78	0.18
	E					100	100-100	333.77	120.00	93.77	10.47	0.01
**Calcified plaque volume - log transformed data**												
Age Sex	ACE	78.68	76.20-82.94	0.00	0.00-40.76	21.32	17.2-23.83	286.02	118.00	50.02		
	**AE**	**78.68**	**76.28-83.19**			**21.32**	**16.81-23.72**	**286.02**	**119.00**	**48.02**	**0.00**	**1.00**
	CE			46.62	40.18-54.86	53.38	45.14-59.82	300.46	119.00	62.46	14.43	0.00
	E					100	100-100	315.65	120.00	75.65	29.63	0.00
ASCVD§ Risk Statin	ACE	80.77	78.25-84.71			19.23	15.83-22.17	294.07	118.00	58.07		
	**AE**	**80.77**	**77.26-83.21**			**19.23**	**16.79-22.74**	**294.07**	**119.00**	**56.07**	**0.00**	**1.00**
	CE			54.69	49.00-61.48	45.31	38.52-51.00	309.04	119.00	71.04	14.97	0.00
	E					100	100-100	330.56	120.00	90.56	36.48	0.00
**Calcified plaque volume - inverse normal transformed data**												
Age Sex	ACE	47.17	44.71-57.96			52.83	42.04-55.34	341.96	118.00	105.96		
	**AE**	**47.17**	**43.81-55.85**			**52.83**	**44.15-56.19**	**341.96**	**119.00**	**103.96**	**0.00**	**1.00**
	CE			26.69	22.96-36.17	73.31	63.83-77.04	346.23	119.00	108.23	4.27	0.04
	E					100	100-100	350.81	120.00	110.81	8.85	0.01
ASCVD§ Risk Statin	ACE	47.82	41.25-54.91	0.00	0.00-50.07	52.18	45.43-58.94	340.68	118.00	104.68		
	**AE**	**47.82**	**41.24-54.83**			**52.18**	**44.17-58.76**	**340.68**	**119.00**	**102.68**	**0.00**	**1.00**
	CE			28.65	22.62-36.80	71.35	63.20-77.38	344.81	119.00	106.81	4.13	0.04
	E					100	100-100	349.99	120.00	109.99	9.31	0.01

[*]A: additive genetic factor, [†]C: common environmental factor, [‡]E: unique environmental factor, §ASCVD: Atherosclerotic Cardiovascular Disease

Table S5: Detailed model information regarding single trait classical twin model of non-calcified plaque volume (n = 196). The most parsimonious models are bolded.

Adjusted for	Estimated parameters	A*	95% CI	C†	95% CI	‡E	95% CI	Model *-2LL*	df	AIC	Difference to full model *-2LL*	Difference to full model *p*
**Non-calcified plaque volume - raw data**												
Age Sex	ACE	77.03	72.70-86.95			22.97	16.22-27.6	444.20	190.00	64.20		
	**AE**	**77.03**	**72.31-83.69**			**22.97**	**16.31-27.69**	**444.20**	**191.00**	**62.20**	**0.00**	**1.00**
	CE			55.06	43.51-60.90	44.94	39.10-56.49	461.73	191.00	79.73	17.53	0.00
	E					100	100-100	497.13	192.00	113.13	52.93	0.00
ASCVD§ Risk Statin	ACE	77.44	70.91-83.89			22.56	16.11-29.09	461.15	190.00	81.15		
	**AE**	**77.44**	**69.14-83.39**			**22.56**	**16.61-30.86**	**461.15**	**191.00**	**79.15**	**0.00**	**1.00**
	CE			55.02	43.90-65.48	44.98	34.52-56.10	476.48	191.00	94.48	15.34	0.00
	E					100	100-100	507.47	192.00	123.47	46.32	0.00
**Non-calcified plaque volume - log transformed data**												
Age Sex	**ACE**	**11.77**	**3.66-17.56**	**82.54**	**76.45-90.09**	**5.7**	**4.64-7.02**	**259.27**	**190.00**	**-120.73**		
	AE	93.81	91.17-94.8			6.19	5.20-7.83	288.64	191.00	-93.36	29.36	0.00
	CE			91.97	90.45-93.79	8.03	6.21-9.55	265.36	191.00	-116.64	6.09	0.01
	E					100	100-100	448.65	192.00	64.65	189.37	0.00
ASCVD§ Risk Statin	**ACE**	**7.47**	**3.94-13.34**	**88.68**	**82.58-91.75**	**3.85**	**3.25-4.90**	**297.67**	**190.00**	**-82.33**		
	AE	95.10	93.74-95.91			4.9	4.09-6.26	337.37	191.00	-44.63	39.70	0.00
	CE			94.61	93.25-95.6	5.39	4.4-6.75	303.15	191.00	-78.85	5.48	0.02
	E					100	100-100	479.41	192.00	95.41	181.74	0.00
**Non-calcified plaque volume - inverse normal transformed data**												
Age Sex	ACE	21.42	0.00-37.22	45.33	36.02-60.14	33.25	27.89-40.51	506.48	190.00	126.48		
	AE	68.57	62.39-74.76			31.43	25.54-37.61	510.11	191.00	128.11	3.62	0.06
	**CE**			**62.07**	**57.63-68.13**	**37.93**	**31.87-42.37**	**507.48**	**191.00**	**125.48**	**1.00**	**0.32**
	E					100	100-100	555.17	192.00	171.17	48.69	0.00
ASCVD§ Risk Statin	ACE	16.07	0.00-37.91	50.09	35.59-59.44	33.84	29.34-42.45	503.03	190.00	123.03		
	AE	67.91	59.97-73.62			32.09	26.38-40.03	507.52	191.00	125.52	4.49	0.03
	**CE**			**62.56**	**55.67-66.70**	**37.44**	**33.30-44.33**	**503.60**	**191.00**	**121.60**	**0.57**	**0.45**
	E					100	100-100	548.02	192.00	164.02	44.99	0.00

*A: additive genetic factor, †C: common environmental factor, ‡E: unique environmental factor, §ASCVD: Atherosclerotic Cardiovascular Disease

Table S6: Detailed model information regarding single trait classical twin model of non-calcified plaque volume excluding the 0-0 pairs (n = 124). The most parsimonious models are bolded.

Adjusted for	Estimated parameters	A[*]	95% CI	C[†]	95% CI	[‡]E	95% CI	Model *-2LL*	df	AIC	Difference to full model *-2LL*	Difference to full model *p*
**Non-calcified plaque volume - raw data**												
Age Sex	ACE	70.40	65.20-84.06	0.00	0.00-67.67	29.60	21.27-38.05	299.11	118.00	63.11		
	**AE**	**70.40**	**62.92-78.42**			**29.60**	**21.58-37.08**	**299.11**	**119.00**	**61.11**	**0.00**	**1.00**
	CE			46.89	33.74-56.64	53.11	43.36-66.26	307.97	119.00	69.97	8.87	0.00
	E					100	100-100	323.37	120.00	83.37	24.26	0.00
ASCVD§ Risk Statin	ACE	73.48	61.99-85.69	0.00	0.00-66.46	26.52	19.33-35.40	309.30	118.00	73.30		
	**AE**	**73.48**	**64.60-80.05**			**26.52**	**19.95-35.40**	**309.30**	**119.00**	**71.30**	**0.00**	**1.00**
	CE			51.24	38.91-61.50	48.76	38.50-61.08	317.76	119.00	79.76	8.46	0.00
	E					100	100-100	335.03	120.00	95.03	25.73	0.00
**Non-calcified plaque volume - log transformed data**												
Age Sex	ACE	61.90	54.09-74.57	2.25	0.00-44.05	35.85	29.00-44.14	286.50	118.00	50.50		
	**AE**	**64.24**	**55.62-71.34**			**35.76**	**28.66-44.38**	**286.50**	**119.00**	**48.50**	**0.00**	**0.95**
	CE			51.87	45.12-60.69	48.13	39.31-54.88	289.73	119.00	51.73	3.23	0.07
	E					100	100-100	309.16	120.00	69.16	22.66	0.00
ASCVD§ Risk Statin	ACE	43.70	20.41-68.13	25.84	0.00-49.70	30.46	25.05-36.96	296.83	118.00	60.83		
	**AE**	**70.36**	**64.67-75.78**			**29.64**	**24.22-35.33**	**297.52**	**119.00**	**59.52**	**0.69**	**0.41**
	CE			59.68	52.91-66.19	40.32	33.81-47.09	299.20	119.00	61.20	2.37	0.12
	E					100	100-100	325.45	120.00	85.45	28.62	0.00
**Non-calcified plaque volume - inverse normal transformed data**												
Age Sex	ACE	51.76	35.53-54.07	0.00	0.00-33.82	58.24	47.19-64.86	343.55	118.00	107.55		
	**AE**	**41.76**	**35.01-51.97**			**58.24**	**48.03-64.99**	**343.55**	**119.00**	**105.55**	**0.00**	**1.00**
	CE			29.45	24.93-38.58	70.55	61.42-75.07	345.26	119.00	107.26	1.71	0.19
	E					100	100-100	350.89	120.00	110.89	7.34	0.03
ASCVD§ Risk Statin	ACE	44.99	40.53-59.68			55.01	46.15-52.78	341.19	118.00	105.19		
	**AE**	**44.99**	**35.65-52.27**			**55.01**	**47.73-64.35**	**341.19**	**119.00**	**103.19**	**0.00**	**1.00**
	CE			32.49	25.13-39.51	67.51	60.49-74.87	342.88	119.00	104.88	1.68	0.19
	E					100	100-100	349.53	120.00	109.53	8.34	0.02

[*]A: additive genetic factor, [†]C: common environmental factor, [‡]E: unique environmental factor, §ASCVD: Atherosclerotic Cardiovascular Disease
